# Magnetic field assisted beam-scanning leaky-wave antenna utilizing one-way waveguide

**DOI:** 10.1038/s41598-019-53431-8

**Published:** 2019-11-14

**Authors:** Lujun Hong, Yun You, Qian Shen, Yazhou Wang, Xing Liu, Hang Zhang, Chiaho Wu, Linfang Shen, Xiaohua Deng, Sanshui Xiao

**Affiliations:** 1 Key laboratory of Poyang Lake Environment and Resource Utilization of Ministry of Education, School of Resources Environmental and Chemical Engineering, Nanchang University, Nanchang, 330031 China; 2 DTU Fotonik, Department of Photonics Engineering, Technical University of Denmark, DK-2800 Kgs. Lyngby, Denmark; 3 Institute of Space Science and Technology, Nanchang University, Nanchang, 330031 China; 4 Department of Applied Physics, Zhejiang University of Technology, Hangzhou, 310023 China; 5 State Key Laboratory of Electronic Thin Films and Integrated Devices, School of Optoelectronic Information, University of Electronic Science and Technology of China (UESTC), Chengdu, 610054 China; 6 School of Marine Science and Technology, Northwestern Polytechnical University, Xi’an, Shanxi 710072 China

**Keywords:** Magneto-optics, Sub-wavelength optics, Nanophotonics and plasmonics

## Abstract

We propose a Leaky-Wave Antenna (LWA) based on one-way yttrium-iron-garnet (YIG)-air-metal waveguide. We first analyze the dispersion of the LWA, showing the one-way feature and the radiation loss. Owing to the unique one-way dispersive property, the beam radiated from the LWA can have very narrow beam width, at the same time having large scanning angle. The main beam angle obtained by full-wave simulation is consistent with our theoretical prediction with the aid of the dispersion. For a given frequency, we can realize continuous beam scanning by varying the magnetic field, where the 3 dB beam width is much narrower than previously demonstrated. Our results pave a new way to realize continuous angle scanning at a fix frequency for modern communications.

## Introduction

Leaky-Wave Antennas (LWAs), first proposed by Hansen *et al*., through opening a narrow slot on a metal waveguide^1^, have been widely used for many applications including radar and satellite communications, and multiplexing devices^2^ due to their advantages of low profile, narrow beam, and low loss. The uniform (quasi-uniform) LWA and periodic-structure-based LWA are the commonly used antennas^3,4^, and they are capable of beam scanning in forward or backward direction, but typically not broadside. In order to overcome this challenge, many efforts have been made by designing different waveguide structures at microwave^5^ and terahertz^6^ frequencies, such as composite right/left-handed (CRLH) transmission line^7^, metamaterial^8^, and spoof surface plasmon polaritons^9,10^. These LWAs can realize continuous scanning from backward, broadside to forward by gradually tuning the operating frequency^11^, but their 3 dB beam width is relatively large. Moreover, the beam scanning property of the LWAs at a fixed frequency has also received much attention^12,13^, and different methods have been applied to obtain fixed-frequency continuous scanning via changing the dc bias voltage^14,15^, showing a large scanning angel of more than 80 degrees^16^. However, most of the LWAs generally suffer from back reflection at the end of waveguide due to the impedance matching, thus limiting the performance with respect to the radiation directivity.

Recently, an attractive and effective way to prevent the end reflection has been proposed by using one-way mode, where the time reversal symmetry were broken by an applied static magnetic field. This concept was first proposed as analogues of quantum Hall edge states in photonic crystals^17^, and such one-way mode can only propagate in one direction and suppress backscattering^18,19^. Afterward, different schemes for realized one-way propagation have been reported^20–26^. The one-way waveguide enables us to deal with the issue of the end reflection in the design of the LWA due to the absence of the backforward-propagating mode. A uniform ferrite-loaded LWA based on one-way mode has been recently proposed^27^. It is capable of both fixed-bias frequency scanning and fixed-frequency bias scanning. However, this ideal model needs a perfect magnetic conductor, which is difficult to be realized in practice.

In this letter, we propose a LWA based on our proposed one-way yttrium-iron-garnet (YIG)-air-metal waveguide^23^ with periodic holes in the metal layer, where static magnetic field (H_0_) is applied. The dispersion of the leaky mode supported by LWA will show the one-way feature, resulting in suppression of the backward mode. The proposed LWA shows the broad scanning angle and narrow 3 dB beam width for the continuous beam scanning by tuning the frequency. More importantly, it exhibits the continuous fixed-frequency beam scanning with broad scanning angle and narrow 3 dB beam width by varying the magnetic field.

## Results

### Dispersion properties of one-way waveguide and LWA

**Figure 1:**
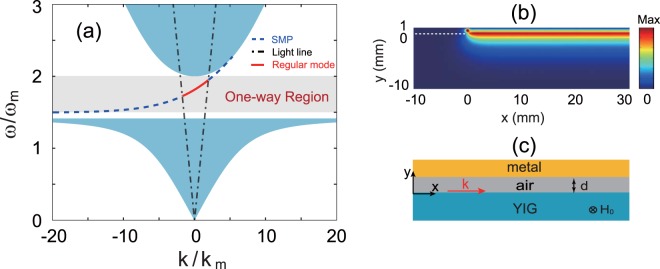
(**a**) Dispersion relations of SMPs (the dashed lines) and regular mode (the solid line) for ${{\rm{H}}}_{0}=1785\,{\rm{G}}$ in the YIG-air-metal structure. The middle shaded area indicates the one-way region for the waveguide, and the other shaded areas indicate the zones of bulk modes in the YIG. (**b**) Simulated electric field amplitudes at $f=9\,{\rm{GHz}}$. (**c**) Schematic of the YIG-air-metal structure under an external magnetic field H_0_. The thickness of the air layer is $d=1\,{\rm{mm}}$.

In order to investigate the proposed LWA, we first analyze the dispersion property of the YIG-air-metal structure, as illustrated in Fig. 1(c). The thickness of the air layer is denoted by *d*, and the thickness of the YIG is assumed to be semi-infinite. With the static magnetic field applied in the −*z* direction, the YIG in the waveguide is gyromagnetic anisotropic with the relative permittivity ${\varepsilon }_{m}\,=\,15$ and (relative) permeability tensor *μ*_*m*_:

1 $${\overleftrightarrow{\mu }}_{m}=[\begin{array}{ccc}{\mu }_{1} & -\,i{\mu }_{2} & 0\\ i{\mu }_{2} & {\mu }_{1} & 0\\ 0 & 0 & 1\end{array}],$$

with ${\mu }_{1}=1+\frac{{\omega }_{m}({\omega }_{0}-iv\omega )}{{({\omega }_{0}-iv\omega )}^{2}-{\omega }^{2}}$ and ${\mu }_{2}=\frac{{\omega }_{m}\omega }{{({\omega }_{0}-iv\omega )}^{2}-{\omega }^{2}}$, where $\omega$ is the angular frequency, ${\omega }_{m}$ is the characteristic circular frequency, ${\omega }_{0}=2\pi \gamma {{\rm{H}}}_{0}$ (*γ* = 2.8 × 10^6^ rad/s/G is the gyromagnetic ratio) is the precession angular frequency, and $v=\frac{\gamma \Delta H}{2\omega }$ (Δ*H* is the resonance linewidth) is the damping coefficient^28^. Such a two-dimensional (2D) waveguide can support both SMPs and the regular mode, and their dispersion relations are given by

2 $${\alpha }_{r}{\mu }_{v}+({\alpha }_{m}+\frac{{\mu }_{2}}{{\mu }_{1}}k)\,\tanh ({\alpha }_{r}d)=0$$

where ${\alpha }_{m}=\sqrt{{k}^{2}-{\varepsilon }_{m}{\mu }_{v}{k}_{0}^{2}}$ with ${\mu }_{v}={\mu }_{1}-{\mu }_{2}^{2}/{\mu }_{1}$ (The Voigt permeability) and ${k}_{0}=\omega /c$ (The free-space wave number), and ${\alpha }_{r}=\sqrt{{k}^{2}-{k}_{0}^{2}}$ (SMPs), while ${\alpha }_{r}=-\,i\sqrt{{k}_{0}^{2}-{k}^{2}}$ for the regular mode^23,26^. The linear term with respect to *k* in Eq. (2) indicates that both modes are non-reciprocal. The dispersion relations for SMPs and the regular mode in the YIG-air-metal structure are numerical calculated, and the results are shown the dashed lines and solid line in Fig. 1(a), respectively. Here, we assume the YIG medium to be lossless ($\Delta H=0\,{\rm{G}}$) with ${\omega }_{m}=10\pi \times {10}^{9}$ rad/s (${f}_{m}=5\,{\rm{GHz}}$), and ${\omega }_{0}$ is set at ${\omega }_{m}$, which corresponds to ${{\rm{H}}}_{0}=1785\,{\rm{G}}$. The thickness of the air layer is fixed as $d=1\,{\rm{mm}}$ to gain large band for the one way propagation. As seen in Fig. 1(a), there exists a one-way propagation band (the middle shaded area) in the bandgap of the magnetized YIG, whose bulk modes (the uppest and lowest shaded areas) are given by ${k}^{2} < {\varepsilon }_{m}{\mu }_{v}{k}_{0}^{2}$. The one-way region ranges from ${\omega }_{sp}$ to ${\omega }_{0}+{\omega }_{m}$, equivalent to the frequency region $[7.5,10]\,{\rm{GHz}}$, where ${\omega }_{sp}={\omega }_{0}+0.5{\omega }_{m}$ is the asymptotic frequency of SMPs at $k\to -\,\infty$. In this region, the modes are allowed to propagate only in the forward direction due to the group velocity of $dw/dk > 0$. To further verify its one-way guiding property, we perform the simulation of wave transmission with the finite element method (FEM) using COMSOL Multiphysics. In the simulation, the metal in the system was assumed to be a perfect electric conductor, and a linear magnetic current source located on the center of the air layer was used to excite wave. Figure 1(b) shows the simulated electric field amplitudes for $f=9\,{\rm{GHz}}$. Evidently, the electromagnetic wave can only propagate in the forward direction as expected.

**Figure 2:**
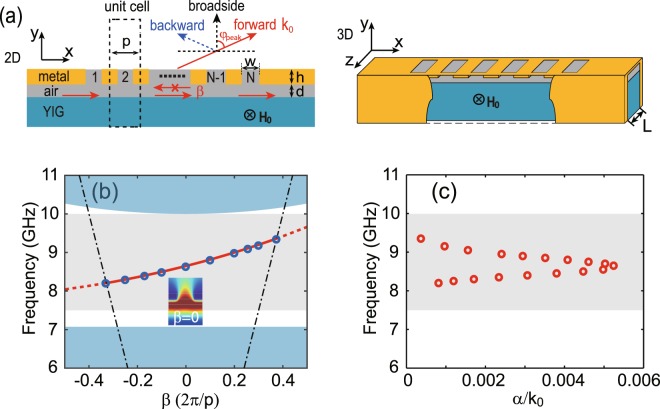
(**a**) Schematic of the 2D (the left panel) and 3D (the right panel) LWA with antenna width of *L* composed by a metallic periodic structure, an air layer, and a substrate with YIG material under an external magnetic field H_0_. The cell number for the structure with the period of *p* is *N*, and the width and depth of the holes are *w* and *h*, respectively. The thickness of the air layer between the periodic structure and YIG is *d*. (**b**) Dispersion relation of the LWA. The solid and dashed lines represent the leaky mode and guided mode, respectively. Circles represent the results for the 3D LWA. The middle shaded area indicates the one-way region for the waveguide, and the dot-dashed lines represent the light lines. The inset shows the electric field distribution at $\beta =0$ and ${{\rm{H}}}_{0}=1785\,{\rm{G}}$. (**c**) The loss for the leaky mode versus the frequency.

Then, we analyze the dispersion property of the proposed LWA, which is formed by the YIG-air-metal waveguide with periodic holes in the metal layer, as illustrated in the left panel of Fig. 2(a). The period of the unit cell is denoted by *p*, and the width and depth of the hole are denoted by *w* and *h*, respectively. This 2D LWA can support the transverse electric (TE) mode whose electric field is polarized along the *z* direction. With respect to a practical device, a 3D LWA, see the right panel in Fig. 2(a), is proposed with the finite width of the antenna, sandwiched by two metal slabs in the z direction. By solving the eigenfrequency problem, the dispersion relation of the LWA was calculated with FEM. Note that the performance of the LWA is strongly dependent on its structural parameters. In this work, our interest focuses on analyzing the dependence of continuous scanning property on the non-structural parameters, such as the frequency and applied magnetic field. As an example, the parameters of the periodic unit are $w=6\,{\rm{mm}}$, $h=1\,{\rm{mm}}$, and $p=12\,{\rm{mm}}$, respectively.

Figure 2(b) shows the dispersion relation of the leaky mode (the solid line) and guiding mode (the dashed lines) of LWA when ${{\rm{H}}}_{0}=1785\,{\rm{G}}$. The leaky mode lies within the light cone (indicated by the dot-dashed lines). In the whole first Brillouin zone, the modal group velocity ($d\omega /d\beta$) is always positive, which indicates the one-way propagation behavior. This is confirmed by the results shown in Fig. 4. The dispersion for the leaky mode lies in the one-way region for the YIG-air-metal waveguide, see the middle shaded area in Fig. 2(b). We also calculate the dispersion relation for the 3D system with the waveguide width of $L=5\,{\rm{mm}}$, and the obtained results (see circles) agree well with those for the 2D system. The loss, associated with the radiation, for the leaky mode is illustrated in Fig. 2(c). Note that in our calculation we do not take the material loss into account. The radiation loss changes significantly when tuning the frequency, and shows the maximum around $\beta =0$. The electric field distribution in the unit cell at $\beta =0$ is also displaced in the inset of Fig. 1(b), indicating the leaky feature. For the given structure parameters, the frequency range of the LWA is $[8.2,9.35]\,{\rm{GHz}}$. The two intersections between the light lines and the dispersion curve imply that we can control the beam radiation angle from −90° to 90° when changing the frequency. Especially, at the frequency of $f=8.64\,{\rm{GHz}}$, *β* becomes almost zero, meaning that this mode can radiate at broadside. As illustrated in Fig. 2(b), the phase of the leaky mode can be continuously changed by tuning the frequency. For our proposed LWA, compared to high-order harmonic, the radiation by the 0th fundamental harmonic is predominant, and the beam angle for the 0th harmonic is given by

3 $${\phi }_{peak}=\arcsin (\beta /{k}_{0})$$

**Figure 3:**
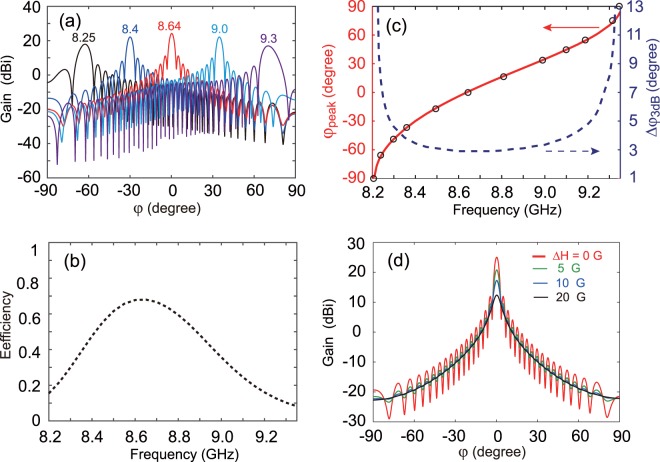
(**a**) Far-field radiation patterns (gain) of the LWA with continuous frequency scanning. Marked is the value of the frequency *f* (in GHz). (**b**) The radiation efficiency of LWA. (**c**) The numerical (solid line) and analytic (circles) results of the radiation angle ${\phi }_{peak}$, and 3 dB beam width $\Delta {\phi }_{3dB}$ (dashed line) as a function of *f*. (**d**) Far-field radiation patterns (gain) for different losses Δ*H* at $f=8.64\,{\rm{GHz}}$. The solid lines from the top to bottom represent Δ*H* = 0, 5, 10, and 20 G, respectively. The applied magnetic field is set to ${{\rm{H}}}_{0}=1785\,{\rm{G}}$.

**Figure 4:**
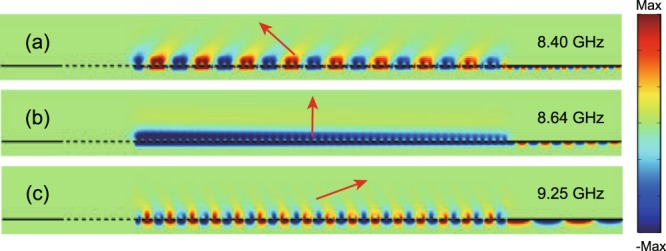
Simulated z-components of the electric fields for the LWA at different frequencies. (**a**) 8.4 GHz, (**b**) 8.64 GHz, and (**c**) 9.25 GHz. The excited source is placed at the center of the air layer in the periodic structure of LWA.

According to Eq. (3), when $-\,{k}_{0}\le \beta  < 0$, the backward beam scanning ($-\,90^\circ \le {\phi }_{peak} < 0^\circ$) can be obtained; whereas the forward beam scanning ($0^\circ  < {\phi }_{peak}\le 90^\circ$) is achieved when $0 < \beta \le {k}_{0}$.

### Frequency scanning LWA

To verify the continuous frequency scanning feature of the LWA, we calculate the electric field distributions and far-field radiation patterns by the full-wave simulation. In the simulation, wave is first coupled into the YIG-air-metal waveguide at its left side, see Fig. 2(a), and when it travels forward within the waveguide, it gradually radiates to the free space through the periodic holes, and the residual wave exits at the right side. The number of the periodic hole, denoted by *N*, usually needs to be sufficiently large to achieve high directivity, and here it is fixed as $N=50$. Figure 3(a) shows the far-field radiation patterns of the LWA for ${{\rm{H}}}_{0}=1785\,{\rm{G}}$ at different frequencies: 8.25, 8.4, 8.64, 9, and 9.3 GHz, see the solid lines from left to right. As expected, the beam radiation angle can be controlled from backward, broadside, to forward direction by changing the frequency. Moreover, the gain of the antenna shows a maximum value of ${G}_{gain}=25\,{\rm{dBi}}$ at $f=8.64\,{\rm{GHz}}$ with the beam angle of ${\phi }_{peak}=0^\circ$, and it gradually decreases in the forward and backward direction when tuning the frequency. Figure 3(b) shows the radiation efficiency of LWA. This result agrees well with the result for the radiation loss shown in Fig. 2(c), and the maximum radiation energy obtained at ${\phi }_{peak}=0^\circ$ is also consistent with the result that the radiation loss is largest when $\beta =0$ at $f=8.64$ GHz. The numerical beam angle ${\phi }_{peak}$ (the solid line) and the 3 dB beam width $\Delta {\phi }_{3dB}={\phi }_{3dB}^{+}-{\phi }_{3dB}^{-}$ (the dashed line) as a function of *f* are displayed in Fig. 3(c), where ${\phi }_{3dB}^{+}$ and ${\phi }_{3dB}^{-}$ are the larger and smaller half-power (−3 dB) points of the main lobe respectively. It can be seen that the LWA has a wide scanning angle with high directivity. The scanning angle is 107.8° for $\Delta {\phi }_{3dB}\le 5^\circ$, and it can reach as high as 143° for $\Delta {\phi }_{3dB}\le 10^\circ$ by changing the frequency from 8.23 GHz to 9.31 GHz. For a large scanning angle, $\Delta {\phi }_{3dB}$ increases significantly because the transverse width of the radiated beam is largely reduced. As an example, ${\phi }_{peak}=-\,51^\circ$ and $\Delta {\phi }_{3dB}=4.8^\circ$ when $f=8.29\,{\rm{GHz}}$, ${\phi }_{peak}=0^\circ$ and $\Delta {\phi }_{3dB}=2.9^\circ$ at $f=8.64\,{\rm{GHz}}$, and ${\phi }_{peak}=56.8^\circ$ and $\Delta {\phi }_{3dB}=4.95^\circ$ at $f=9.21\,{\rm{GHz}}$. Moreover, we also calculate the values of ${\phi }_{peak}$ with Eq. (2) for various frequencies, as seen the circles in Fig. 3(c). Obviously, this analytic result is in good agreement with those obtained by our full-wave simulations.

Besides, we systematically analyze the radiation pattern of LWA on different losses Δ*H*. It can be found from Fig. 3(d) that with the increase of Δ*H* from 0 to 20 G, the radiation angle almost remains unchanged and the 3 dB beam width increases slightly; meanwhile, the gain of LWA decreases from 25 dBi to 12.3 dBi. As an example, ${\phi }_{peak}=0^\circ$, $\Delta {\phi }_{3dB}=3.1^\circ$, and ${G}_{gain}=20.7\,{\rm{dBi}}$ for $\Delta H=5\,{\rm{G}}$ at the frequency of $f=8.64\,{\rm{GHz}}$; while ${\phi }_{peak}=0.05^\circ$, $\Delta {\phi }_{3dB}={5.6}^{\circ }$, and ${G}_{gain}=12.3\,{\rm{dBi}}$ for $\Delta H=20\,{\rm{G}}$. To further clearly show the guiding property of LWA, the simulated z-components of electric fields at different frequencies: *f* = 8.4, 8.64, and 9.25 GHz are displaced in Fig. 4(a–c), respectively. Evidently, the electromagnetic wave can only propagate forward as expected when placing a line current source in the periodic structure of LWA, which is in a good agreement with the one-way property of the leaky mode in Fig. 2(b). More importantly, the wave radiates toward different directions from backward to forward when increasing the frequency, as shown in Fig. 4. Therefore, the LWA exhibits continuous frequency-scanning from the backward to forward directions with the large scanning angle and narrow 3 dB beam width. It should be noted that in the absence of the applied magnetic field, the LWA will lose the continuous scanning property because it does not support any guided wave.

### Fixed-frequency scanning LWA

**Figure 5:**
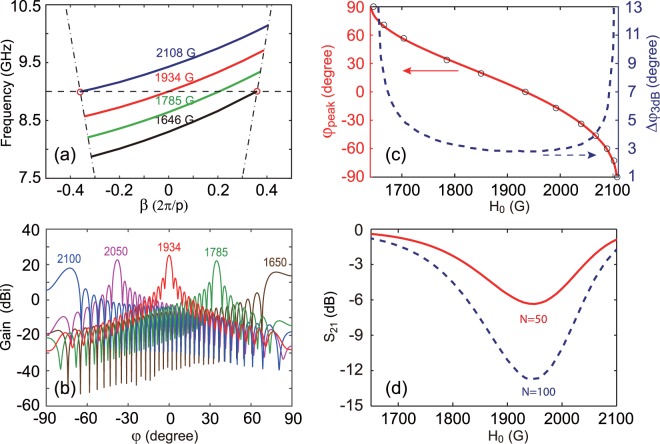
(**a**) Dispersion relations of the leaky mode of LWA for various H_0_. The horizontal dashed line shows the fixed frequency of *f*_0_ = 9 GHz for the LWA with continuous magnetic field scanning. (**b**) Far-field radiation patterns (gain) of the LWA with continuous magnetic field scanning. (**c**) The numerical (solid line) and analytic (circles) results of ${\phi }_{peak}$, and $\Delta {\phi }_{3dB}$ (dashed line) versus H_0_. (**d**) *S*_21_ versus H_0_ of the LWA for $N=50$ (solid line) and $N=100$ (dashed line).

For the LWA discussed above, the value of the magnetic field is fixed at ${{\rm{H}}}_{0}=1785\,{\rm{G}}$. Compared to the frequency-scanning LWA, the frequency-independent LWA is preferable for applications in modern communication systems. Here, the property of the proposed LWA strongly depends on the applied magnetic field H_0_ due to the usage of the magneto-optical material. To investigate the influence of H_0_ on the dispersion of the LWA, we calculate the dispersion relations for the leaky modes at various H_0_, as shown in Fig. 5(a). It can be seen that the dispersion curves shift up when increasing H_0_. We emphasize that when tuning H_0_ the dispersion for the leaky mode always lie in the one-way region for the waveguide. Here we choose a fixed frequency of ${f}_{0}=9\,{\rm{GHz}}$ as an example, see the horizontal dashed line in Fig. 5(a). When tuning the magnetic field from 1646 G to 2108 G, we can control *β* changing from $\beta ={k}_{0}$ to −*k*_0_, see the two circles in Fig. 5(a). This implies that the beam angle for the LWA in principle can be realized from −90° to 90° by tuning the magnetic field for the given frequency. Especially, when ${{\rm{H}}}_{0}=1934\,{\rm{G}}$, *β* becomes zero, meaning that this mode can radiate at the broadside for the fixed-frequency LWA.

**Figure 6:**
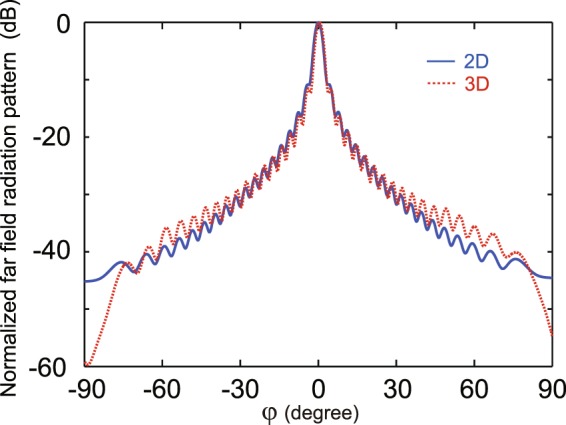
The normalized far-field radiation patterns of the 2D LWA (solid line) and 3D LWA with the waveguide width of $L=5\,{\rm{mm}}$ (dashed line) for ${{\rm{H}}}_{0}=1934\,{\rm{G}}$ and $\Delta H=5\,{\rm{G}}$ in the xy plane. The working frequency is *f* = 9 GHz.

To verify the continuous fixed-frequency scanning feature of the LWA, the far field radiation patterns at different H_0_ values for ${f}_{0}=9\,{\rm{GHz}}$ are shown in Fig. 5(b). It is clearly seen that the continuous scanning behavior of the LWA can be realized by tuning the magnetic field from 1650 G to 2100 G. The antenna gain is found to be maximum at ${{\rm{H}}}_{0}=1934\,{\rm{G}}$ with the beam angle of ${\phi }_{peak}=0^\circ$, and it gradually decreases in the forward and backward direction when tuning the magnetic field, which is similar to that for the frequency scanning shown in Fig. 3(a). ${\phi }_{peak}$ and $\Delta {\phi }_{3dB}$ versus H_0_ are illustrated as the solid and dashed lines in Fig. 5(c), respectively. The results for ${\phi }_{peak}$ are in a good agreement with those obtained by Eq. (3) for various H_0_, as seen the circles in Fig. 5(c). The LWA has a narrow 3 dB beam width of $\Delta {\phi }_{3dB}\le 5^\circ$ in a large scanning angle of 110.9°, when the magnetic field varies from 1703 G to 2081 G. The 3 dB beam width is almost insensitive to the magnetic field in the range of [1703, 2081] G. The scanning angle can also reach as high as 145° for $\Delta {\phi }_{3dB}\le 10^\circ$ when tuning H_0_ from 1660 G to 2100 G. As an example, ${\phi }_{peak}=55.9^\circ$ and $\Delta {\phi }_{3dB}=5^\circ$ at ${{\rm{H}}}_{0}=1703\,{\rm{G}}$, ${\phi }_{peak}=-\,55^\circ$ and $\Delta {\phi }_{3dB}=4.85^\circ$ when ${{\rm{H}}}_{0}=2081\,{\rm{G}}$, and ${\phi }_{peak}=0^\circ$ and $\Delta {\phi }_{3dB}=2.8^\circ$ at the point of ${{\rm{H}}}_{0}=1934\,{\rm{G}}$. Besides, we also calculate the S-parameters of the LWA for two different *N* values: $N=50$ (the solid line) and 100 (the dashed line), as illustrated in Fig. 5(d). The transmission coefficient *S*_21_ is found to be minimum around ${{\rm{H}}}_{0}=1934\,{\rm{G}}$, which is consistent with the results shown in Fig. 5(b). Note that in our proposed LWA, *S*_21_ (dB) decreases linearly with *N* and *S*_11_ is always zero due to the suppression of the reflected waves. We also evaluate the radiation pattern for the 3D model, see the right panel of Fig. 2(a), when we take the loss ($\Delta H=5\,{\rm{G}}$) into account. The width of 3D waveguide is 5 mm in the *z* direction. It can be observed from Fig. 6 that the normalized far-field radiation patterns of 2D and 3D LWAs in the xy plane are in good agreement.

Table 1 shows the comparison of performances between the proposed one-way waveguide based LWA and several published LWAs. Liu *et al*.^7^, and Paulotto *et al*.^11^ reported different kinds of frequency scanning LWAs, whose maximum beam-scanning ranges are 40° and 89°, respectively. Different fixed-frequency scanning LWAs are reported in^12,14^, whose maximum beam-scanning ranges are 21° and 104°, respectively. Compared with these LWAs, the proposed LWAs exhibit larger scanning angle (>107°) and smaller 3 dB beam width (<5°) for both frequency scanning and fixed-frequency scanning. Moreover, this structure is relatively easy to be realized in practice, when comparing to the previous ferrite-loaded LWA^27^.

**Table 1:** Comparison of the proposed LWA and other published LWAs.

Frequency scanning LWA			Fixed-frequency scanning LWA		
Refs	Maximum scanning angle	3 dB angle	Refs	Maximum scanning angle	3 dB angle
^7^	40° (−20°, +20°)	unknown	^12^	21° (+9°, +30°)	unknown
^11^	89° (−42°, +47°)	>15°	^14^	104° (−49°, +55°)	>15°
**This work**	107.8° (−51°, +56.8°)	<5°	**This work**	110.9° (−55°, +55.9°)	<5°
	143° (−70.9°, +72.1°)	≤10°		145.2° (−72.5°, +72.7°)	≤10°

## Conclusions

In conclusion, we have proposed a LWA based on one-way YIG-air-metal waveguide with periodic holes under a static external magnetic field. The dispersion and radiation properties of the LWA have been analyzed, showing that the leaky mode supported by the LWA exhibits one-way feature. With the aid of one-way waveguide, the radiated beam by the LWA can have narrow 3 dB beam width, while at the same time having large scanning angle. The main beam angle obtained by the full-wave simulations are confirmed by our theoretical prediction. More importantly we have realized continuous beam scanning by varying the magnetic field at a fixed frequency. Our results demonstrated here show a promising way to realize continuous angle scanning for modern communications.

## Methods

The dispersion curves, and radiation efficiency of LWA were calculated by commercial FEM software (COMSOL). The electric field distribution and far field radiation pattern of the LWA were simulated by COMSOL. The dispersion curves of the modes in YIG-air-metal structure was calculated by Matlab.
